# Association between PM_2.5_ Exposure and All-Cause, Non-Accidental, Accidental, Different Respiratory Diseases, Sex and Age Mortality in Shenzhen, China

**DOI:** 10.3390/ijerph16030401

**Published:** 2019-01-31

**Authors:** Junfang Cai, Chaoqiong Peng, Shuyuan Yu, Yingxin Pei, Ning Liu, Yongsheng Wu, Yingbin Fu, Jinquan Cheng

**Affiliations:** 1 National Institute of Environmental Health and Related Product Safety, Chinese Center for Disease Control and Prevention, Beijing 100021, China; caijunfang@nieh.chinacdc.cn; 2 Shenzhen Center for Disease Control and Prevention, Shenzhen 518055, China; pcq@szcdc.net (C.P.); shuyuanyu2008@163.com (S.Y.); liun@szcdc.net (N.L.); cdc@szcdc.net (Y.W.); fuyingbin0320@163.com (Y.F.); 3 CFETP, Chinese Center for Disease Control and Prevention, Beijing 100050, China; peiyingxin@hotmail.com

**Keywords:** air pollution, mortality, cause-specific, sex, age, generalized additive model, time-series

## Abstract

*Background*: China is at its most important stage of air pollution control. Research on the association between air pollutants and human health is very important and necessary. The purpose of this study was to evaluate the association between PM_2.5_ concentrations and residents’ mortality and to compare the effect of PM_2.5_ on the different diseases, accidental deaths, sex or age of residents from high polluted areas with less polluted areas. *Methods*: The semi-parametric generalized additive model (GAM) with Poisson distribution of time series analysis was used. The excess risk (ER) of mortality with the incremental increase of 10 µg/m^3^ in PM_2.5_ concentration was calculated. Concentration-response relationship curves and autocorrelation between different lags of PM_2.5_ were also evaluated. *Results*: PM_2.5_ exposure was significantly associated with the mortality of residents. The strongest ERs per 10 µg/m^3^ increase in PM_2.5_ were 0.74% (95% CI: 0.11–1.38%) for all-cause, 0.67% (95% CI: 0.01–1.33%) for non-accidental, 1.81% (95% CI: 0.22–3.42%) for accidental, 3.04% (95% CI: 0.60–5.55%) for total respiratory disease, 6.38% (95% CI: 2.78–10.11%) for chronic lower respiratory disease (CLRD), 8.24% (95% CI: 3.53–13.17%) for chronic obstructive pulmonary disease (COPD), 1.04% (95% CI: 0.25–1.84%) for male and 1.32% (95% CI: 0.46–2.19%) for elderly. Furthermore, important information on the concentration-response relationship curves was provided. *Conclusions*: PM_2.5_ can increase the risk of residents’ mortality, even in places with less air pollution and developed economy in China.

## 1. Introduction

Air pollution is harmful to human health [1,2]. Exposure to air pollution increases mortality and morbidity and is an important cause of the global disease burden [3]. Research has shown that ambient air pollution had become the fourth biggest threat to the health of the Chinese people in 2010 [4]. Among atmospheric pollutants, airborne fine particulate matter (containing fine particulates often measured as particulate matter ≤2.5 μm in aerodynamic diameter; PM_2.5_) is one that was consistently associated with adverse human health and of great concern to the general public [5,6,7]. 

China had undergone a period of reform and opening-up and extensive economic development. China’s economy had been growing rapidly for decades. At present, it is in the transition period from extensive development to refined development. With the rapid growth of the economy, China, which accounts for one fifth of the world’s population (according to China’s sixth census, the population of the Chinese mainland accounts for 19.27 percent of the world’s population), had become one of the countries with the most serious air pollution in the world [8,9,10]. The average annual nationwide concentration of PM_2.5_ in 272 cities in China from 2013 to 2015 was 56 µg/m^3^ [11]. Information from the air quality status report of China National Environmental Monitoring Centre indicates that the average concentration of PM_2.5_ in 338 cities in China in January 2016, January 2017 and January 2018 was 68 µg/m^3^, 78 µg/m^3^ and 64 µg/m^3^, respectively [12].

The Chinese government is paying more and more attention to the health effects of air pollution. China was at its most important stage of air pollution control. In 2013, the State Council of China issued the Air Pollution Prevention and Control Action Plan (APPCAP) to alleviate severe air pollution and associated adverse health effects in China. In 2018, the report of the nineteenth National Congress of the Communist Party of China regarded pollution control as one of the three major battles [13]. China’s air quality has improved in recent years. Annual average concentrations of PM_2.5_ decreased by 33.3%, PM_10_ by 27.8%, sulfur dioxide (SO_2_) by 54.1%, and carbon monoxide (CO) 28.2% between 2013 and 2017 in the 74 key cities in China [14]. Air pollution control has been effective in China. The concentration of major air pollutants is decreasing, but the overall situation of air pollution is still grim.

Since China has only begun monitoring PM_2.5_ concentration in 2013 and there is a lack of standardized long-term monitoring data, so most previous studies are based on short-term or intermittent data [14,15,16,17,18,19]. Recent studies had focused mainly on the national level and the overall study from multiple cities or the evaluation of pollution control measures [11,20]. This study focuses on the association between PM_2.5_ concentration and mortality among residents in Shenzhen, China, exploring the effects of PM_2.5_ on different diseases, sex or age. This would potential contribute to research on the impact of PM_2.5_ to human health in a region characterized by slight pollution.

**Figure 1:**
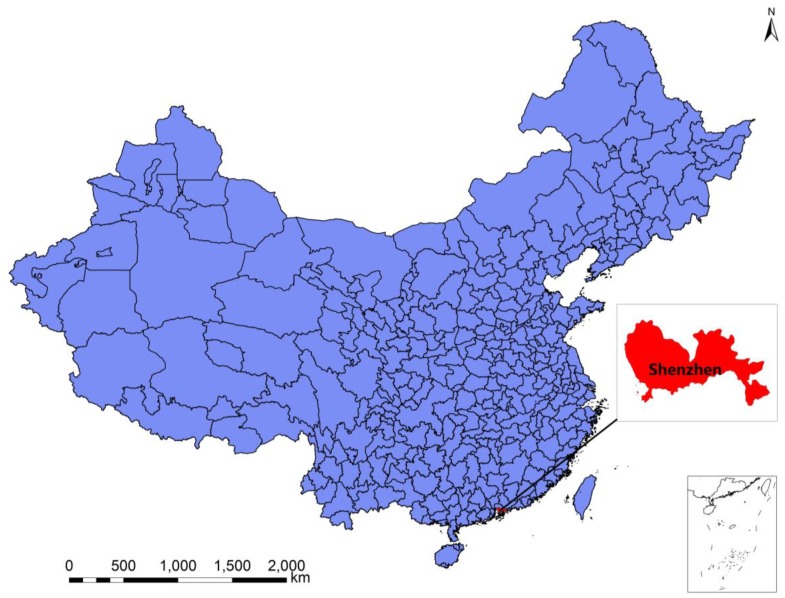
Location of Shenzhen in China.

Shenzhen is situated in southeastern China, 113°46–114°37 E and 22°27–22°52 N, the area of 1991.64 km^2^ (Figure 1). The year-end permanent population in 2013, 2014 and 2015 was 1062.89, 1077.89 and 1137.87 (×10**^5^** persons), respectively [21,22]. Shenzhen has a subtropical monsoon climate with long summers and short winters, mild climate, abundant sunshine and abundant rainfall. The average annual temperature is 23 °C, the historical extreme maximum temperature is 39 °C, the historical extreme minimum temperature is 0 °C, the average temperature in January is the lowest (15 °C), the average temperature in July is the highest (29 °C), the average annual sunshine is 1838 h and the average annual precipitation is 1935.8 mm. Shenzhen, with a developed economy, is China’s first special economic zone and a window for China’s reform and opening-up. It is one of the three major national financial centers in China. In 2015, agriculture, light industry and heavy industry represented 0.1, 22.8 and 77.1 of the economic output, respectively (gross output value of industry and agriculture = 100). In Shenzhen, the annual average concentration of PM_2.5_ was 35 µg/m^3^ in 2013–2015. Air quality was better than in many parts of China’s interior (China National Ambient Air Quality Standards 35 µg/m^3^ for annual mean at residential areas) [23]. The PM_2.5_ pollution level was still higher than many American and European countries and the WHO standard (U.S. National Ambient Air Quality Standards 15 µg/m^3^ for annual mean, The Global Air Quality Guidelines set by World Health Organization 10 µg/m^3^ for annual mean). This study aims to assess the association between PM_2.5_ concentration and residents’ mortality and to know the effect of PM_2.5_ on the different disease, accidental, sex or age residents in a place with less air pollution and a developed economy in China.

## 2. Materials and Methods 

### 2.1. Materials

#### 2.1.1. Mortality Data 

Data on residents’ mortality in Shenzhen during 1 January 2013–31 December 2015 (a total of 1095 days) were obtained from Shenzhen Center for Disease Control and Prevention, Shenzhen Public Security Bureau and Shenzhen Funeral Home. Each record includes as variables identity card number, name, sex, age, date of birth, date of death, the main cause of death and the main cause of death ICD coding. Data for the three agencies were compared in accordance with identity card number and name. Data for the three agencies were compared in accordance with identity card number and name. There were 41,815 entries after excluding repetitive data. After grouping the daily mortality of residents and sorting and screening the data, data were encoded as non-accidental mortality (A00–R99), accidental mortality (Initials were S, T, V, W, X, Y), total respiratory disease mortality (J00–J99), chronic lower respiratory disease (CLRD) (J40–J47), chronic obstructive pulmonary disease (COPD) (J44) according to the International Classification of Diseases Revision 10 (IDC-10). Elderly people means those older than 65 years old; younger people means those younger than 65 years old. There were no deaths due to the flu in the database. 

#### 2.1.2. Air Pollution Data

Exposure estimates of the air pollutants in this study included PM_2.5_, particulate matter with particle size below 10 microns (PM_10_), sulfur dioxide (SO_2_), nitrogen dioxide (NO_2_), carbon monoxide (CO) and ozone (O_3_). We obtained exposure data, which were daily air pollutant monitoring data, provided by the seven national-level monitoring sites of the Shenzhen Environmental Monitoring Station, covering the period from 1 January 2013–31 December 2015. The air pollution PM_2.5_, PM_10_, SO_2_, NO_2_ and CO data were based on the means of 24-h average concentration values at the seven sites in Shenzhen. In China, the concentration of O_3_ is reported as an 8-h moving average concentration. We used the 8-h moving average concentration for O_3_ to ensure comparisons with Chinese Ambient Air Quality Standards. Monitoring of PM_2.5_, PM_10_, SO_2_, NO_2_, CO and O_3_ were done in accordance with the Chinese Ambient Air Quality Standards (GB 3095-2012) and the Chinese Technical Regulation for Ambient Qir Quality Assessment (HJ 663-2013) [23,24]. The concentration values of PM_2.5_, PM_10_, SO_2_, NO_2_ and CO 24-h average concentration and O_3_ 8-h moving average concentration represented the exposure city-level.

#### 2.1.3. Meteorological Data

Meteorological data were daily routine monitoring data released by Shenzhen Weather Center, covering the period of 1 January 2013–31 December 2015, including daily average temperature, daily average relative humidity (RH), daily average atmospheric pressure, daily average wind speed, Daily rainfall and sunshine. 

### 2.2. Methods

#### 2.2.1. Basic Description

Mortality, air pollutant and meteorological data are verified to be the not normally distributed. The data were described by mean, standard deviation (SD), range (minimum–maximum) and median (Q1, Q3) (Q1, median, Q3 are equal to the 25%, 50% and 75% number of all values in the sample arranged from small to large, respectively) in order to show the data more clearly. 

#### 2.2.2. Autocorrelation Analysis 

The autocorrelation analysis of daily PM_2.5_ concentration from 1 January 2013–31 December 2015 (1095 days) was conducted. The autocorrelation function and partial autocorrelation function of daily PM_2.5_ concentration series were calculated.

#### 2.2.3. Analysis of Time Series

Mortality data were combined into the daily sex-specific, age-specific and cause-specific mortality through grouping, screening and summary. The air pollutant variables, PM_2.5_ and CO, were based on the means of 24-h average concentration values. The air pollutant O_3_ was based on the means of 8-h sliding average concentration values. Multiple time series chart were produced with time on the horizontal axis, while cause-specific mortality, sex-specific mortality, age-specific mortality, concentrations of pollutants (all-cause mortality, non-accidental mortality, accidental mortality, total respiratory disease mortality, CLRD mortality, COPD mortality, male mortality, female mortality, elder mortality, younger mortality, PM_2.5,_ CO and O_3_) on the vertical coordinate axes. 

#### 2.2.4. Generalized Additive Models, GAM

In the first stage, the basic model was constructed. Mortality data correspond to a Poisson distribution [25,26]. Analysis of time series with a generalized additive model (GAM) based on Poisson distribution were used to establish the model [11,26,27,28,29,30,31,32]. The dependent variables was linked with the independent variables through log transformation [33,34]. Several confounders were introduced in the models: (1) Influence of long-term trend, seasonal trend, day of the week (DOW), public holiday (PH), daily average temperature and daily average relative humidity were controlled [35,36]. The long-term trend, daily average temperature and daily average relative humidity were adjusted by cubic regression spline function [37]. Seasonal trend, DOW and public holiday were adjusted by dummy variables. (2) Sensitivity analysis was carried out. The best parameters were established basing on the unbiased risk estimate (UBRE, UBRE is a readjustment standard of Akaike information criterion (AIC)) value and previous literature [38,39]. We adjusted the degrees of freedom (ʊ) in the smoothness of long-term trend (1–14/year) and meteorological conditions (temperatures and relative humidity degrees of freedom from 1 to 7 in smooth functions) alternately. For the basic regression model, we ultimately used seven degrees of freedom (ʊ) per year for the time variable, three ʊ for temperature variable, and three ʊ for humidity variable.

In the second stage, the single pollutant model was constructed. PM_2.5_ concentration entered into the model as independent variables. Single-day lag models underestimate the cumulative effect of pollutants on mortality [40]. Therefore, we built both single-day lag and multi-day lag models. We used single lags of 0, 1, 2, 3, 4 and 5 days (lag0–lag5) to explore the lag pattern in the effects. PM_2.5_ concentrations with moving averages from day 0 to day 1–4 prior to the mortality (lag01–lag04) were also included in the model, respectively. 

In the third stage, the two-pollutant models were constructed. The higher the correlation coefficient of PM_2.5_ and other factors, the higher the probability of collinearity. We did not control for SO_2_ and NO_2_ in the regression models because PM_2.5_ was highly correlated with SO_2_ or NO_2_ [41]_._ And the previous literature showed that simultaneously entering SO_2_ (or NO_2_) into the models can bring unstable parameter estimates when the pollutants involved suffer high inter-correlation [42]. The correlation between CO and PM_2.5_, and O_3_ and PM_2.5_, were low [41,43]. CO and O_3_ had an impact on human health [44,45]. CO and O_3_ were controlled respectively in the regression models in order to avoid the effect of collinearity and control confounders. The independent impact of PM_2.5_ on the mortality of residents was examined. CO and O_3_ entered the model respectively. Their lags and the moving average concentration days (lag0–lag5, lag01–lag04) were consistent with that of PM_2.5_. Two-pollutant models were constructed, respectively. The daily average concentration of pollutants PM_2.5_, CO and O_3_ were used as independent variables to enter the model.

Finally, to roughly evaluate the potential public health impact of our estimates, we calculated the excess risk (ER, ER = ($e^{\beta \times 10}-1$)) in cause-specific mortality attributable to a 10 µg/m^3^ reduction in the daily PM_2.5_ level. ER and its 95% CI value of the cause-specific mortality were calculated using regression coefficients and standard errors. Quantitative analysis of the short-term effect of PM_2.5_ upon residents’ mortality was conducted [36].

Furthermore, we conducted stratification analyses by age and sex for exploring the potential individual-level effect modifiers of PM_2.5_ using the previously mentioned models.

The determined model expression was as follows:

*Log*[*E(y_t_)*] = *βX_t_* + *s*(*time,* ʊ_1_) + *as.factor*(*season*) + *as.factor*(*dow*) + *as.factor*(*holiday*) + *s*(*temperature,* ʊ_2_) + *s*(*humidity,* ʊ_3_) + *α*

where *y_t_* represents the number of mortality at day *t*; *E*(*y_t_*) represents the expected number of mortality at day *t*; *β* is the linear regression coefficients estimated by GAM; X*_t_* indicates the concentration of pollutants at day *t*; *s* is nonparametric smoothing function; ʊ_1_ is the degree of freedom for adjusting long-term trend in non-parametric function; ʊ_2_ and ʊ_3_ are the degree of freedom for adjusting non-parametric smoothing function daily average temperature and daily average relative humidity; *season* is the dummy variable for season; *dow* and holiday are the dummy variable for day of the week and holiday respectively used to control the short-term fluctuations in the number of daily residents’ mortality; *α* is the residual error [37].

Mortality, air pollution and meteorological data were not missing. In order to analyze the lag effect, there were missing values of pollutant concentration. The corresponding record of the missing value was deleted. If *p* < 0.05, the effect estimates were considered statistically significant, and borderline significant if *p* < 0.10 [46]. 

#### 2.2.5. Analysis of Concentration-Response Relationship 

In each single-day lag (lag0–lag5), the strongest effect of PM_2.5_ concentration upon residents’ different cause mortality was chosen to make concentration-response relationship figures. The concentration-response relationship between PM_2.5_ concentration and all-cause mortality, non-accidental mortality, accidental mortality, total respiratory disease mortality, CLRD mortality, COPD mortality, male mortality, female mortality, elder mortality, younger mortality were shown, respectively.

The software R3.2.0 (open source software, Auckland, New Zealand) was used for statistical analysis. A generalized additive model was constructed by using the penalty spline function of gam in the mgcv package.

## 3. Results

### 3.1. Basic Information of Mortality Residents 

A total of 41,815 residents died in Shenzhen in 2013–2015. On average, there were 38 deaths per day; the number of residents deaths was in range of 10–77 people every day; the average age of death including all causes was 58 years old; the age range was 0–113 years old; 26,567 males and 15,243 females died in 2013–2015; giving a male-female ratio of roughly 1.7:1.35, 467 people died due to non-accidental causes, accounting for 85% of the total number of deaths; 10–71 people died due to non-accidental causes every day; the average age of non-accidental death was 61 years old, and the age range was 0–109 years old. 6348 people died due to accidents, accounting for 15% of the total number of deaths; 0–17 people died due to accidents every day; the average age of accidental death was 42 years old, and the age range was 0–113 years old. 2534 people died due to respiratory diseases, accounting for 6% of the total number of deaths; 0–11 people died due to respiratory diseases every day; the average age of respiratory diseases death was 71 years old, and the age range was 0–107 years old; 1049 people died because of CLRD, accounting for 41% of the total respiratory disease deaths; 0–7 people died due to CLRD every day; the average age of CLRD death was 76 years old, and the age range was 0–102 years old. 646 people died because of COPD, accounting for 26% of the total respiratory disease deaths; 0–5 people died due to COPD every day; the average age of COPD death was 79 years old, and the age range was 7–102 years old. See Table 1.

### 3.2. Information of Air Pollutants and Meteorological Factors

In 2013–2015, the annual average PM_2.5_ concentration in Shenzhen was 35 µg/m^3^; Days when the PM_2.5_ 24-h average concentration exceeded the national grade 1 criterion of China (concentration limit < 35 µg/m^3^) was 458 days and the national grade 2 criterion of China (concentration limit < 75 µg/m^3^) 58 days [23]. PM_2.5_ showed a downward trend in three years. See Table 2. 

### 3.3. Autocorrelation Analysis of PM_2.5_ Concentration 

**Figure 2:**
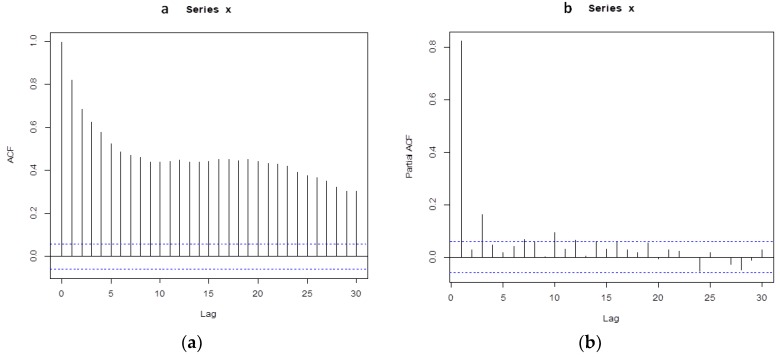
The autocorrelation function and partial autocorrelation function of daily PM_2.5_ concentration. ACF = autocorrelation function; Partial ACF = partial autocorrelation function. (**a**) The autocorrelation function of daily PM_2.5_ concentration. (**b**) The partial autocorrelation function of daily PM_2.5_ concentration.

The autocorrelation analysis of daily PM_2.5_ concentration for 2013–2015 years was carried out. The autocorrelation function and partial autocorrelation function showed that the PM_2.5_ concentration series had a significant autocorrelation. See Figure 2.

### 3.4. Time Series Chart on Residents’ Mortality versus Air Pollutants Concentration

**Figure 3:**
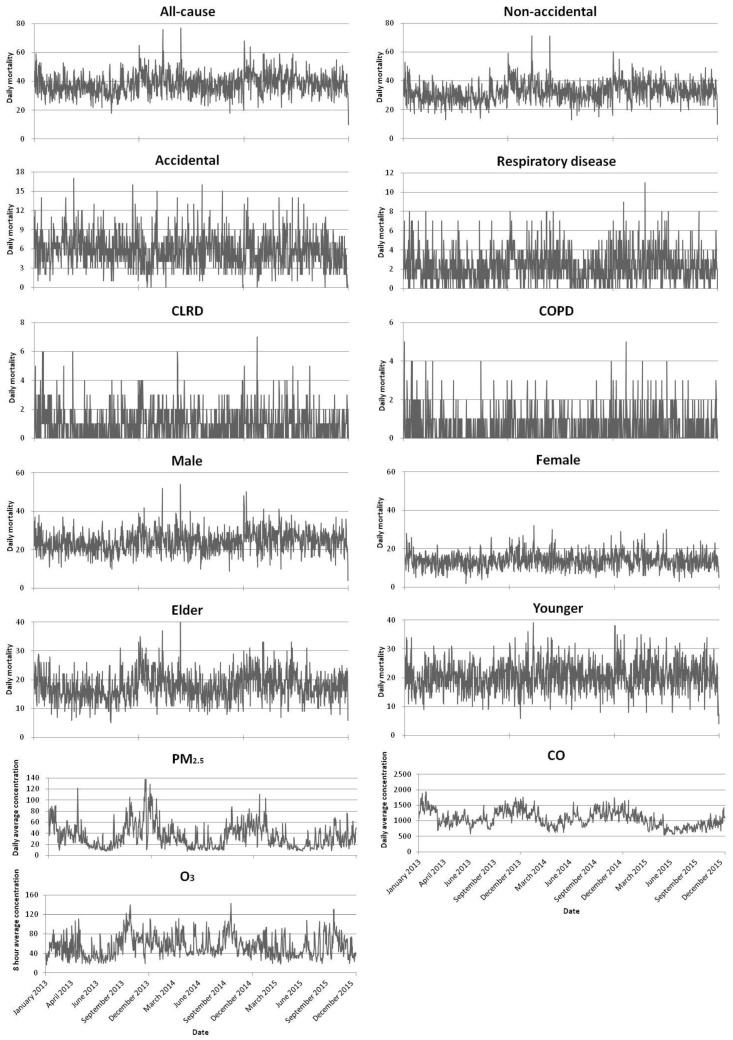
Time Series Chart on Residents’ Mortality versus Air Pollutants Concentration Day by Day in Shenzhen (2013–2015). CLRD = chronic lower respiratory disease; COPD = chronic obstructive pulmonary disease; Elder = age is greater than or equal to 65 years old; Younger = age is less than 65 years old.

Time series diagram of resident mortality and air pollutant concentration for 2013–2015 years displayed that the variation trend of all-cause mortality, non-accidental, accidental, total respiratory disease, male and elder mortality counts and that of PM_2.5_ concentration basically matched; both peaked in winter (November, December and January), and both declined in summer (May, June and July). CO and O_3_ had also seasonal trend. See Figure 3.

### 3.5. Analysis of Generalized Additive Model, GAM

Single pollutant model and two-pollutant models were used to calculate the RR value and its 95% CI lag0–lag5, lag01–lag04 of PM_2.5_ 24-h average concentration versus mortality in Shenzhen (Supplementary Material, Figure S1). 

The effects of PM_2.5_ concentration on all-cause mortality, non-accidental mortality, accidental mortality, total respiratory disease mortality, CLRD mortality, COPD mortality, male mortality, female mortality, elder mortality and younger mortality without controlling other pollutants and after controlling CO or O_3_ see Table 3.

#### 3.5.1. Effects of PM_2.5_ Concentration on All-Cause Mortality

Without controlling for other pollutants, PM_2.5_ concentration of lag1, lag2, lag5 on the all-cause mortality had significant effects, of which lag2 was the most significant. For every 10 µg/m^3^ PM_2.5_ concentration of lag2 rose, the ER of total residents’ mortality was 0.74% (95% CI: 0.11–1.38%). PM_2.5_ moving average concentration (lag01–lag04) on the all-cause mortality had significant effects, of which lag02 was the most significant. For every 10 µg/m^3^ PM_2.5_ concentration of lag02 rose, the ER of total residents’ mortality was 0.39% (95% CI: 0.14–1.73%).

After controlling CO, the results showed that PM_2.5_ concentration of lag0–lag5 on the all-cause mortality had significant effects, of which lag2 was the most significant. For every 10 µg/m^3^ PM_2.5_ concentration of lag2 rose, the ER of total residents’ mortality was 1.00% (95% CI: 0.30–1.70%). PM_2.5_ moving average concentration (lag01–lag04) on the all-cause mortality had significant effects, of which lag02 was the most significant. For every 10 µg/m^3^ PM_2.5_ concentration of lag02 rose, the ER of total residents’ mortality was 1.26% (95% CI: 0.40–2.12%).

After controlling O_3_, the results showed that PM_2.5_ concentration of lag1, lag2 and lag5 on the all-cause mortality had significant effects, of which lag5 was the most significant. For every 10 µg/m^3^ PM_2.5_ concentration of lag5 rose, the ER of total residents’ mortality was 1.07% (95% CI: 0.37–1.77%). PM_2.5_ moving average concentration (lag02–lag04) on the all-cause mortality had significant effects, of which lag04 was the most significant. For every 10 µg/m^3^ PM_2.5_ concentration of lag04 rose, the ER of total residents’ mortality was 1.12% (95% CI: 0.09–2.16%). 

#### 3.5.2. Effects of PM_2.5_ Concentration on Non-Accidental Mortality

Without controlling other pollutants, PM_2.5_ concentration of lag2 and lag5 on the non-accidental mortality had significant effects, of which lag5 was the most significant. For every 10 µg/m^3^ PM_2.5_ concentration of lag5 rose, the ER of non-accidental residents’ mortality was 0.67% (95% CI: 0.01–1.33%). PM_2.5_ moving average concentration (lag02–lag04) on the non-accidental mortality had significant effects, of which lag02–lag04 were marginally significant. 

After controlling CO, the results showed that PM_2.5_ concentration of lag0–lag2, lag4–lag5 on the non-accidental mortality had significant effects, of which lag4 was the most significant. For every 10 µg/m^3^ PM_2.5_ concentration of lag4 rose, the ER of non-accidental residents’ mortality was 0.89% (95% CI: 0.16–1.62%). PM_2.5_ moving average concentration (lag01–lag04) on the non-accidental mortality had significant effects, of which lag04 was the most significant. For every 10 µg/m^3^ PM_2.5_ concentration of lag04 rose, the ER of non-accidental residents’ mortality was 1.24% (95% CI: 0.25–2.23%). 

After controlling O_3_, the results showed that PM_2.5_ concentration of lag2 and lag5 on the non-accidental mortality had significant effects, of which lag5 was the most significant. For every 10 µg/m^3^ PM_2.5_ concentration of lag5 rose, the ER of non-accidental residents’ mortality was 1.10% (95% CI: 0.35–1.86%). PM_2.5_ moving average concentration (lag03–lag04) on the non-accidental mortality had significant effects, of which lag04 was the most significant. For every 10 µg/m^3^ PM_2.5_ concentration of lag04 rose, the ER of non-accidental residents’ mortality was 1.11% (95% CI: 0.01–2.23%).

#### 3.5.3. Effects of PM_2.5_ Concentration on Accidental Mortality

Without controlling other pollutants, PM_2.5_ concentration of lag2 on the accidental mortality had significant effects. For every 10 µg/m^3^ PM_2.5_ concentration of lag2 rose, the ER of accidental residents’ mortality was 1.81% (95% CI: 0.22–3.42%). PM_2.5_ moving average concentration (lag01–lag04) on the accidental mortality had significant effects, of which lag02 was the most significant. For every 10 µg/m^3^ PM_2.5_ concentration of lag02 rose, the ER of accidental residents’ mortality was 2.26% (95% CI: 0.30–4.25%). 

After controlling CO, the results showed that PM_2.5_ concentration of lag1 and lag2 on the accidental mortality had significant effects, of which lag2 was the most significant. For every 10 µg/m^3^ PM_2.5_ concentration of lag2 rose, the ER of accidental residents’ mortality was 2.16% (95% CI: 0.41–3.94%). PM_2.5_ moving average concentration (lag02–lag04) on the accidental mortality had significant effects, of which lag02 was the most significant. For every 10 µg/m^3^ PM_2.5_ concentration of lag02 rose, the ER of accidental residents’ mortality was 2.40% (95% CI: 0.27–4.56%). 

After controlling O_3_, the results showed that PM_2.5_ moving average concentration of lag02 on the accidental mortality had significant effects. For every 10 µg/m^3^ PM_2.5_ concentration of lag02 rose, the ER of accidental residents’ mortality was 2.01% (95% CI: −0.26–4.33%). 

#### 3.5.4. Effects of PM_2.5_ Concentration on Total Respiratory Disease Mortality

Without controlling other pollutants, PM_2.5_ concentration of lag3–lag5 on the total respiratory disease mortality had significant effects, of which lag3 was the most significant. For every 10 µg/m^3^ PM_2.5_ concentration of lag2 rose, the ER of total respiratory disease mortality was 3.04% (95% CI: 0.60–5.55%). After controlling CO, PM_2.5_ concentration of lag3 on total respiratory disease mortality had significant effect. For every 10 µg/m^3^ PM_2.5_ concentration of lag3 rose, the ER of total residents’ respiratory disease mortality was 2.62% (95% CI: −0.04–5.34%). After controlling O_3_, PM_2.5_ concentration of lag3 on the total respiratory disease mortality had a significant effect. For every 10 µg/m^3^ PM_2.5_ concentration of lag3 rose, the ER of total residents’ respiratory disease mortality was 4.17% (95% CI: 1.40–7.02%). PM_2.5_ moving average concentration (lag01–lag04) on the total respiratory disease mortality had no significant effects. 

#### 3.5.5. Effects of PM_2.5_ Concentration on CLRD Mortality

Without controlling other pollutants, PM_2.5_ concentration of lag2, lag3 and lag4 on CLRD mortality had significant effects, of which lag3 was the most significant. For every 10 µg/m^3^ PM_2.5_ concentration of lag3 rose, the ER of CLRD mortality was 6.38% (95% CI: 2.78–10.11%). PM_2.5_ moving average concentration (lag03–lag04) on CLRD mortality had significant effects, of which lag04 was the most significant. For every 10 µg/m^3^ PM_2.5_ concentration of lag04 rose, the ER of CLRD mortality was 6.05% (95% CI: 1.01–11.35%). 

After controlling CO, the results showed that PM_2.5_ concentration of lag3 on CLRD mortality had significant effects. For every 10 µg/m^3^ PM_2.5_ concentration of lag3 rose, the ER of CLRD mortality was 4.40% (95% CI: 0.50–8.44%). PM_2.5_ moving average concentration (lag01–lag04) on CLRD mortality had no significant effect. 

After controlling O_3_, the results showed that PM_2.5_ concentration of lag2–lag4 on CLRD mortality had significant effects, of which lag3 was the most significant. For every 10 µg/m^3^ PM_2.5_ concentration of lag3 rose, the ER of CLRD mortality was 9.71% (95% CI: 5.60–13.97%). PM_2.5_ moving average concentration (lag03–lag04) on CLRD mortality had significant effects, of which lag03 was the most significant. For every 10 µg/m^3^ PM_2.5_ concentration of lag03 rose, the ER of CLRD mortality was 8.26% (95% CI: 2.66–14.16%).

#### 3.5.6. Effects of PM_2.5_ Concentration on COPD Mortality

Without controlling other pollutants, PM_2.5_ concentration of lag2–lag5 on COPD mortality had significant effects, of which lag3 was the most significant. For every 10 µg/m^3^ PM_2.5_ concentration of lag3 rose, the ER of COPD mortality was 8.24% (95% CI: 3.53–13.17%. PM_2.5_ moving average concentration (lag03 and lag04) on COPD mortality had significant effects, and lag03 and lag04 were all marginally significant. 

After controlling CO, the results showed that PM_2.5_ concentration of lag3 on COPD mortality had significant effects. For every 10 µg/m^3^ PM_2.5_ concentration of lag3 rose, the ER of COPD mortality was 6.52% (95% CI: 1.43–11.86%). PM_2.5_ moving average concentration (lag01–lag04) on COPD mortality had no significant effect. 

After controlling O_3_, the results showed that PM_2.5_ concentration of lag2–lag5 on COPD mortality had significant effects, of which lag3 was the most significant. For every 10 µg/m^3^ PM_2.5_ concentration of lag3 rose, the ER of COPD mortality was 13.01% (95% CI: 7.53–18.77%). PM_2.5_ moving average concentration (lag03–lag04) on COPD mortality had significant effects, of which lag04 was the most significant. For every 10 µg/m^3^ PM_2.5_ concentration of lag04 rose, the ER of COPD mortality was 11.15% (95% CI: 3.18–19.74%). 

#### 3.5.7. Effects of PM_2.5_ Concentration on Male Mortality

Without controlling other pollutants, PM_2.5_ concentration of lag2 and lag5 on the male mortality had significant effects, of which lag2 was the most significant. For every 10 µg/m^3^ PM_2.5_ concentration of lag2 rose, the ER of male residents’ mortality was 1.04% (95% CI: 0.25–1.84%). PM_2.5_ moving average concentration (lag01–lag04) on the male mortality had significant effects, of which lag02 was the most significant. For every 10 µg/m^3^ PM_2.5_ concentration of lag02 rose, the ER of male residents’ mortality was 1.22% (95% CI: 0.23–2.22%). The effect of PM_2.5_ moving average concentration on male mortality was more obvious.

After controlling CO, the results showed that PM_2.5_ concentration of lag0–lag2 on the male mortality had significant effects, of which lag2 was the most significant. For every 10 µg/m^3^ PM_2.5_ concentration of lag2 rose, the ER of male residents’ mortality was 1.09% (95% CI: 0.23–1.97%). PM_2.5_ moving average concentration (lag01–lag04) on the male mortality had significant effects, of which lag02 was the most significant. For every 10 µg/m^3^ PM_2.5_ concentration of lag02 rose, the ER of male residents’ mortality was 1.40% (95% CI: 0.34–2.48%).

After controlling O_3_, the results showed that PM_2.5_ concentration of lag2, lag3 and lag5 on the male mortality had significant effects, of which lag2 was the most significant. For every 10 µg/m^3^ PM_2.5_ concentration of lag2 rose, the ER of male residents’ mortality was 1.09% (95% CI: 0.19–1.99%). PM_2.5_ moving average concentration (lag02–lag04) on the male mortality had significant effects, of which lag04 was the most significant. For every 10 µg/m^3^ PM_2.5_ concentration of lag04 rose, the ER of male residents’ mortality was 1.45% (95% CI: 0.19–2.72%).

#### 3.5.8. Effects of PM_2.5_ Concentration on Female Mortality

Without controlling other pollutants, PM_2.5_ concentration of lag5 on the female mortality had significant effects. For every 10 µg/m^3^ PM_2.5_ concentration of lag5 rose, the ER of female residents’ mortality was 0.94% (95%CI: −0.05–1.93%). 

After controlling CO, the results showed that PM_2.5_ concentration of lag1, lag3–lag5 on the female mortality had significant effects, of which lag5 was the most significant. For every 10 µg/m^3^ PM_2.5_ concentration of lag5 rose, the ER of female residents’ mortality was 1.62% (95% CI: 0.52–2.73%). PM_2.5_ moving average concentration (lag03–lag04) on the female mortality had significant effects, of which lag04 was the most significant. For every 10 µg/m^3^ PM_2.5_ concentration of lag04 rose, the ER of female residents’ mortality was 1.47% (95% CI: 0.01–2.96%).

After controlling O_3_, the results showed that PM_2.5_ concentration of lag5 on the female mortality had significant effects. For every 10 µg/m^3^ PM_2.5_ concentration of lag5 rose, the ER of female residents’ mortality was 1.39% (95% CI: 0.26–2.53%). 

#### 3.5.9. Effects of PM_2.5_ Concentration on Elderly Mortality

Without controlling other pollutants, PM_2.5_ concentration of lag1–lag5 on the elder mortality had significant effects, of which lag5 was the most significant. For every 10 µg/m^3^ PM_2.5_ concentration of lag5 rose, the ER of elder residents’ mortality was 1.32% (95% CI: 0.46–2.19%). PM_2.5_ moving average concentration (lag01–lag04) on the elder mortality had significant effects, of which lag04 was the most significant. For every 10 µg/m^3^ PM_2.5_ concentration of lag04 rose, the ER of elder residents’ mortality was 1.57% (95% CI: 0.38–2.78%).

After controlling CO, the results showed that PM_2.5_ concentration of lag0–lag5 on the elder mortality had significant effects, of which lag5 was the most significant. For every 10 µg/m^3^ PM_2.5_ concentration of lag5 rose, the ER of elder residents’ mortality was 1.32% (95% CI: 0.37–2.28%). PM_2.5_ moving average concentration (lag01–lag04) on the elder mortality had significant effects, of which lag04 was the most significant. For every 10 µg/m^3^ PM_2.5_ concentration of lag04 rose, the ER of elder residents’ mortality was 1.82% (95% CI: 0.55–3.11%). 

After controlling O_3_, the results showed that PM_2.5_ concentration of lag1, lag2, lag4 and lag5 on the elder mortality had significant effects, of which lag5 was the most significant. For every 10 µg/m^3^ PM_2.5_ concentration of lag5 rose, the ER of elder residents’ mortality was 1.74% (95% CI: 0.75–2.74%). PM_2.5_ moving average concentration (lag02–lag04) on the elder mortality had significant effects, of which lag04 was the most significant. For every 10 µg/m^3^ PM_2.5_ concentration of lag04 rose, the ER of elder residents’ mortality was 1.53% (95% CI: 0.10–2.99%).

#### 3.5.10. Effects of PM_2.5_ Concentration on Younger Mortality

Without controlling other pollutants or after controlling O_3_, PM_2.5_ concentration on the younger mortality had non-significant effects. After controlling CO, the results showed that PM_2.5_ moving average concentration lag02 on the younger mortality had significant effects. For every 10 µg/m^3^ PM_2.5_ concentration of lag02 rose, the ER of younger residents’ mortality was 0.99% (95% CI: −0.18%–2.18%). 

### 3.6. Concentration-Response Relationship 

**Figure 4:**
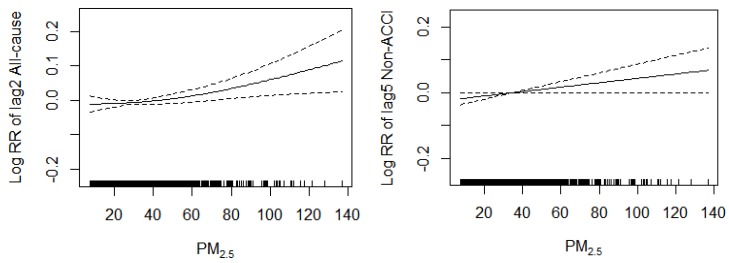

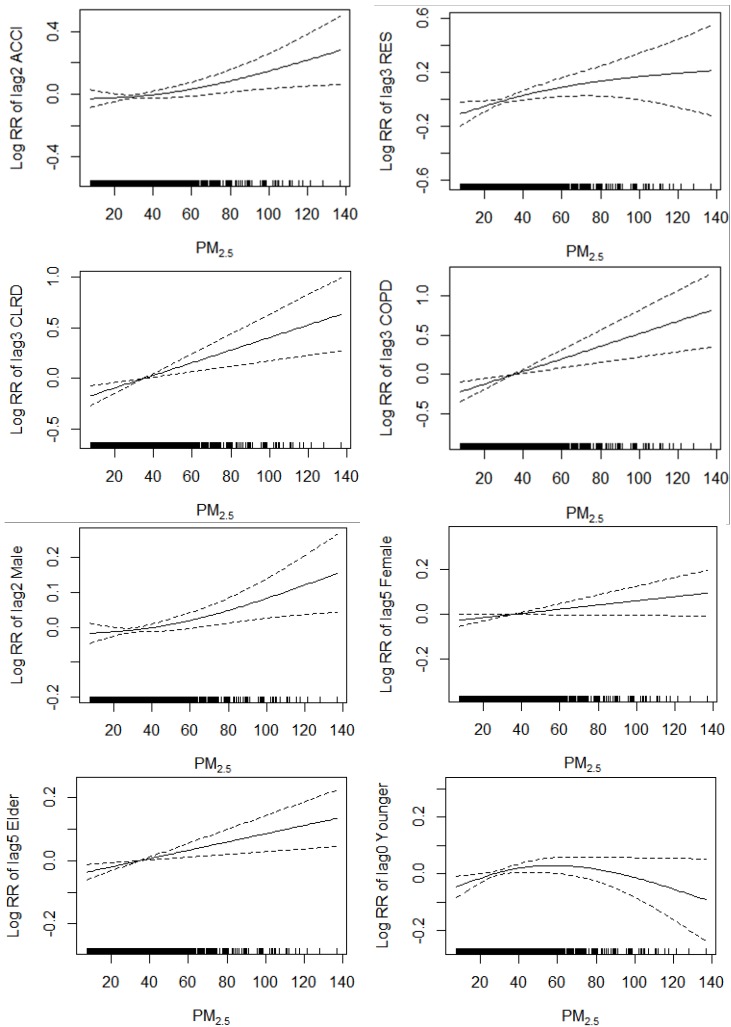
Association between the strongest effect among each single-day lag (lag0–lag5) of residents’ mortality and PM_2.5_ concentration (µg/m^3^) considered in the generalized additive model. The solid line stands for fitting curve and intermittent lines stand for their 95% CI. RR is relative risk; Non-ACCI = non-accidental mortality; ACCI = accidental mortality; RES = total respiratory disease mortality; CLRD = chronic lower respiratory disease; COPD = chronic obstructive pulmonary disease; Elder = age is greater than or equal to 65 years old; Younger = age is less than 65 years old.

The lag day with the strongest effect of PM_2.5_ concentration on the different cause mortality of residents was selected among each single-day lag (lag0–lag5), and the concentration-response figure was drawn. The shape of the concentration-response curve was non-linear for several causes of death examined. See Figure 4 for the concentration-response relationship between PM_2.5_ 24-h average concentration and all-cause mortality, non-accidental mortality, accidental mortality, total respiratory disease mortality, CLRD mortality, COPD mortality, male mortality, female mortality, elderly mortality and younger mortality. 

According to the diagrams, based on the control of long-term and season trend of residents’ mortality, effect of DOW, public holiday, daily average temperature and daily average RH, the relative risk (RR) of all-cause mortality, non-accidental mortality, accidental mortality, total respiratory disease mortality, CLRD mortality, COPD mortality, male mortality, female mortality and elder mortality increased along with the increase in PM_2.5_ 24-h average concentration except for younger mortality. The concentration-response curve for PM_2.5_ (lag0) and younger mortality was slightly non-linear with a sharp slope upward at less than 40 µg/m^3^, a leveling at 40–70 µg/m^3^ and a moderate slope downward with much wider confidence intervals at greater than 70 µg/m^3^.

## 4. Discussion

This study aimed to explore the association between PM_2.5_ and mortality in a place in China with less air pollution and a developed economy. It showed that PM_2.5_ exposure was significantly associated with residents’ mortality. The association for mortality from respiratory causes (particularly from CLRD and COPD) was robust and appeared to be stronger than for either all-cause mortality or non-accidental causes. Association for accidental causes of mortality was greater than for non-accidental causes. CLRD or COPD causes of mortality were greater than for total respiratory causes. There also being lag effects. However, there is no significant association between PM_2.5_ exposure and younger mortality. Results of this study were consistent with the previous literature on the relationship between air pollution and mortality [1,47,48]. 

According to the global data meta-analysis for all-cause mortality, a 10 µg/m^3^ increment in PM_2.5_ was associated with a 1.04% (95% CI: 0.52–1.56%) increase in the risk. The risk of all-cause mortality in North America, other parts of the Americas, Europe and the Western Pacific region increased 0.94% (95% CI: 0.73–1.16%), 2.08% (95% CI: 1.60–2.56%), 1.23% (95% CI: 0.45–2.01%) and 0.25% (95% CI: 0.06–0.44%), respectively [49]. Our results were slightly below the global level, but were beyond that of Western Pacific region. Noteworthy was that our estimate was similar to a pooled estimate (0.94%) in North America. Dai et al. reported that a 10 µg/m^3^ increment in the PM_2.5_ 2 days moving average concentration was associated with a 1.18% (95% CI: 0.93–1.44%) increase in the risk of all-cause mortality, which was similar to our results (0.93%) [1]. Kan et al. reported that in Shanghai the strongest effect was 0.36% (95% CI: 0.11–0.61%) [17]. This was lower than our results (lag02, 0.93%). Venners et al. reported that no significant associations were found between PM_2.5_ and any cause of mortality [19]. 

Our study showed that PM_2.5_ exposure was significantly associated with non-accidental mortality. This was the same with the study of NIH-AARP diet and health cohort by Thurston et al. (HR = 1.03; 95% CI: 1.00–1.05), survivors of myocardial infarction cohort study by Tu et al. (HR = 1.22; 95% CI: 1.03–1.45), a Canadian national-level cohort study by Crouse et al. (HR = 1.15; 95% CI: 1.13–1.16), in the 2001 Canadian Census Health and Environment Cohort study by Pinault et al. ( HR = 1.18; 95% CI: 1.15–1.21) and the Canadian community health survey cohort study by Pinault et al. (HR = 1.26; 95% CI: 1.19–1.34) [48,50,51,52,53]. For a 10 µg/m^3^ increase of PM_2.5_ concentrations, we estimated a 0.67% increase for non-accidental mortality, which was higher than a recent multicity study (0.22%) [11]. Our findings confirm the discovery that the associations were stronger in cities with lower PM_2.5_ levels or higher temperatures [11,54]. Our result was lower than study by Lee et al. which showed that each 10 µg/m^3^ increase in PM_2.5_ (mean lag0 and lag1) was associated with a 1.56% (1.19, 1.94) increase in daily non-accidental mortality, but was higher than study by Baxter et al. [55,56]. Weichenthal et al. reported that PM_2.5_ was not associated with non-accidental mortality based on the Agricultural Health study cohort [57].

We also observed stronger associations between PM_2.5_ exposure and mortality from accidental causes. The ER of accidental mortality was 1.81% (95% CI: 0.22–3.42%) while PM_2.5_ concentration of lag2 rose by every 10 µg/m^3^. PM_2.5_ moving average concentration (lag01–lag04) on the accidental mortality had also significant effects. It suggested that serious air pollution could increase the risk of accidents and aggravate the depressive symptoms of people. In the future, perhaps more attention should be paid to this aspect.

At the global level, a 10 µg/m^3^ increment in PM_2.5_ was associated with a 1.51% (95% CI: 1.01–2.01%) increase in the risk of total respiratory disease mortality. The risk of total respiratory disease mortality in North America, other parts of the Americas, Europe and the Western Pacific region respectively increased 1.39% (95% CI: 0.62–2.16%), 0.88% (95% CI: −1.88–3.71%), 3.81% (95% CI: 0.57–7.16%) and 1.49% (95% CI: 0.04–2.96%) [49]. Our results were slightly below the results of Europe, but exceeded those of North America, other parts of the Americas and the Western Pacific region. For a 10 µg/m^3^ increase of PM_2.5_ concentrations, we estimated a 3.04% increase for respiratory mortality, which was higher than a multicity study (0.29%) [11]. For every 10µg/m^3^ increase in 2-day averaged PM_2.5_ concentration, Dai, et al. reported a 1.71% (95% CI: 1.06–2.35%) increase in respiratory death, and Kan, et al. reported a 0.95% (95% CI: 0.16–1.73%) increase of respiratory mortality [1,17]. Our result (3.04%) was beyond the study of the two results. Pinault et al. reported that each 10 μg/m^3^ increase in exposure was associated with increased risks of respiratory disease mortality (HR  =  1.52; 95 % CI: 1.26–1.84) [53]. PM_2.5_ exposure was not statistically significant associated with respiratory mortality (HR = 1.05; 95% CI: 0.98–1.13) reported by Thurston et al. (2016) [48]. 

Our study showed that PM_2.5_ concentration had a significant positive correlation with CLRD mortality. The strongest ER per 10 µg/m^3^ increase in PM_2.5_ were 6.38% (95% CI: 2.78–10.11%) for CLRD. Hao et al. reported that the association between ambient PM_2.5_ and CLRD mortality was positive but statistically insignificant (RR: 1.07, 95% CI: 0.99–1.14) [58]. 

For a 10 µg/m^3^ increase of PM_2.5_ concentrations, the strongest effect we estimated an 8.24% increase for COPD mortality, which was higher than a multicity study (0.38%) [11]. We observed higher ERs for COPD mortality compared to ER for non-accidental causes of death. The results were the same with the study by Pinault et al. [52]. Li et al. reported that the association between PM_2.5_ concentration and death from COPD was 2.5% (95% CI: 1.5–3.5%) [59]. The results of Li et al. were lower than in our study. Pinault et al. reported that each 10 μg/m^3^ increase in exposure was associated with increased risks of COPD mortality (HR  =  1.40; 95 % CI: 1.09–1.80) [53]. Research demonstrated that a higher risk of acute exacerbation of COPD was associated with present-day PM_2.5_ exposure [60]. Atkinson et al. reported that association between PM_2.5_ and death from COPD was not statistically significant, which was different from our result [49].

Our findings showed that PM_2.5_ was positively associated with male mortality. The result was same with the study by Thurston et al. (HR = 1.03; 95% CI: 1.00–1.06) [48]. Pinault et al. reported an analysis that each 10 μg/m^3^ increase in exposure was associated with increased risks of male mortality (HR  =  1.34; 95 % CI: 1.24–1.46) [53].

Our findings showed that PM_2.5_ exposure was not significantly associated with increased risk of female mortality, but it is noteworthy that after controlling CO or O_3_, there was a significant correlation between PM_2.5_ and female mortality. The results were same as the study by Thurston et al. (HR = 1.02; 95% CI: 0.98–1.06) [48]. Another study by Villeneuve et al. showed that PM_2.5_ exposure was significantly associated with female mortality [61]. Pinault et al. reported that each 10 μg/m^3^ increase in exposure was associated with increased risks of female mortality (HR  =  1.18; 95 % CI: 1.09–1.28) [53].

Our findings showed that PM_2.5_ was significantly associated with elderly mortality, which were similar to an estimate (1.05%) of the study by Alessandrini et al. [62]. Shi et al. reported that PM_2.5_ was associated with increased mortality. 2.14% (95% CI: 1.38–2.89%) increases was estimated for 10 µg/m^3^ increase in lag01 exposure among elder people in the New England area [2]. In our study, lag04 was the most significant. For every 10 µg/m^3^ PM_2.5_ concentration of lag04 rose, the ER of elder mortality was 1.57% (95% CI: 0.38–2.78%). the results of Shi et al. were higher than ours. A study of the Hong Kong elderly population by Qiu et al. showeda statistically significant association of PM_2.5_ exposure over 0–6 day lags with all natural mortality and the overall cumulative effect of PM_2.5_ over 0–30 lag days was 3.44% (95% CI: 0.30–6.67%) increase in all natural mortality [63]. PM_2.5_ exposure was also significantly associated with elderly mortality (HR = 1.03; 95% CI: 1.00–1.06 and HR = 1.14; 95% CI: 1.07–1.22) in the reports by Thurston et al. and by Wong et al. [48,64]. Research demonstrated that a higher risk of acute exacerbation of COPD associated with present-day PM_2.5_ exposure, especially in the elderly [60].

Our study found that PM_2.5_ exposure was not statistically significantly associated with younger mortality, which was the same as the study by Thurston et al. (HR = 1.00; 95% CI: 0.95–1.05) [48]. There was very little research on the association between PM_2.5_ and young people [49].

As shown by the time series figures, the variation tendency of daily residents’ mortality and the daily average PM_2.5_ concentration were basically matched. According to the concentration-response relationship figures, RR of residents’ all-cause mortality, non-accidental, accidental, total respiratory disease, CLRD, COPD, male, female and elder mortality increased along with the increase in PM_2.5_ 24-h average concentration, except for female. Both the time series chart and the concentration-response relationship figures indicated that PM_2.5_ could affect the death of the residents.

During 2013–2015 in Shenzhen, the mean age at death was 58 years old, but the respiratory-caused death was 71 years old, which was younger than China’s average life expectancy (74.83 years old) [9]. One of the possible reasons might be that although having a high-standard living and belonged to the economically developed areas, Shenzhen is a city of immigrants, which has a lot of people coming from all over the country to earn a living. These immigrants were basically young people, so the average age of Shenzhen residents is much younger than many other areas of China [41]. In Shenzhen, the mortality rate was 1.28 per thousand while the national mortality rate was 7.11 per thousand in 2015 [21,22].

From different places in the world, the reported results were not exactly the same. On one hand, it might be relevant to the factors of regions, air-pollution levels, main sources of pollutants and PM_2.5_ components. On the other hand, different adjustments of confounding factors and different measurement methods of exposure in the course of study could also affect the results. Evidence for small study bias in single-city mortality studies was found [49]. Multi-city population-based epidemiological studies of short-term PM_2.5_ exposures and mortality have observed heterogeneity in risk estimates between cities [65]. They found that cities with larger homes, more heating degree days, a higher percentage of home heating with oil had significantly higher health effect estimates, while cities with more gas heating had significantly lower health effect estimates. According to Fang et al., weather conditions could affect health effect estimates [66]. Also, Vodonos et al. found that PM_2.5_ mean exposures of less than 10 µg/m^3^ were associated with higher mortality effect estimates [54]. Atkinson et al. thought that reasons for heterogeneity in effect estimates in different regions of the world require further investigation [49].

Data on residents’ mortality was collected from Shenzhen CDC, Shenzhen Public Security Bureau and Shenzhen Funeral Home in this study, which has the authority to represent the death of local residents. Steady results of this study were based on the data analysis of 2013–2015 for three consecutive years. Several factors that could affect residents’ mortality, like DOW, PH, seasonal trend, long-term trend, daily average humidity and temperature, were controlled in model in this study. In conclusion, this study objectively analyzed the relationship between PM_2.5_ concentration and mortality.

This study, however, also has some limitations. The locally-specific factors that could have effects on correlations between pollutant exposure and mortality refer to PM chemical composition, smoking status, socioeconomic factors, existing emission sources, etc. are not contained in this study, thus needs further researches.

## 5. Conclusions

According to the results of this study, although Shenzhen PM_2.5_ annual average concentration was 35 µg/m^3^, corresponding to a slight pollution level in China, PM_2.5_ still increased the number of all-cause mortality, non-accidental, accidental, total respiratory disease, CLRD, COPD, male and elderly mortality of residents. Therefore, it is recommended that residents were still need to use personal protection in polluted weather, especially those with respiratory diseases, such as CLRD and COPD. The government still needs to strengthen the governance of air pollution despite being a place with less air pollution in China. 

## Figures and Tables

**Table 1:** Basic information of mortality residents in Shenzhen (2013–2015).

Category	Total Deaths	Percent among Total Deaths (%)	Mean (SD)	Range	Median (Q1, Q3)
**Health effects**					
All-cause mortality					
2013	13,126	100	36 (7)	18–59	35 (31, 40)
2014	14,116	100	39 (8)	18–77	39 (33, 43)
2015	14,573	100	40 (7)	10–68	40 (35, 44)
2013–2015	41,815	100	38 (8)	10–77	38 ( 33, 43)
Non-accidental mortality (A00–R99)					
2013	10,846	83	30 (6)	13–53	29 (25, 34)
2014	12,071	86	33 (7)	13–71	33 (28, 37)
2015	12,550	86	34 (7)	10–60	35 (30, 39)
2013–2015	35,467	85	32 (7)	10–71	32 (27, 37)
Accidental mortality (S, T, V, W, X, Y)					
2013	2278	17	6 (3)	1–17	6 (4, 8)
2014	2045	14	6 (3)	0–16	5 (4, 7)
2015	2023	14	6 (3)	0–14	5 (4, 7)
2013–2015	6348	15	6 (3)	0–17	6 (4, 7)
Respiratory disease mortality (J00–J99)					
2013	745	6	2 (2)	0–8	2 (1, 3)
2014	883	6	2 (2)	0–8	2 (1, 3)
2015	906	6	2 (2)	0–11	2 (1, 3)
2013–2015	2534	6	2 (2)	0–11	2 (1, 3)
CLRD mortality (J40–J47)					
2013	319	2	1 (1)	0–6	1 (0, 1)
2014	384	3	1 (1)	0–6	1 (0, 2)
2015	346	2	1 (1)	0–7	1 (0, 1)
2013–2015	1049	3	1 (1)	0–7	1 (0, 2)
COPD mortality (J44)					
2013	192	1	1 (1)	0–5	0 (0, 1)
2014	217	2	1 (1)	0–4	0 (0, 1)
2015	237	2	1 (1)	0–5	0 (0, 1)
2013–2015	646	2	1 (1)	0–5	0 (0, 1)
Sex					
Male					
2013	8258	63	23 (5)	10–38	22 (19, 26)
2014	8881	63	24 (6)	9–54	24 (20, 27)
2015	9428	65	26 (6)	4–50	26 (22, 29)
2013–2015	26,567	64	24 (6)	4–54	24 (20, 28)
Female					
2013	4868	37	13 (4)	2–28	13 (11, 16)
2014	5232	37	14 (4)	5–32	14 (11, 17)
2015	5143	35	14 (4)	3–30	14 (11, 17)
2013–2015	15,243	36	14 (4)	2–32	14 (11, 17)
Sex-Unknown					
2013	0	0	0 (0)	0–0	0 (0, 0)
2014	3	0	0 (0)	0–1	0 (0, 0)
2015	2	0	0 (0)	0–1	0 (0, 0)
2013–2015	5	0	0 (0)	0–1	0 (0, 0)
Age (years)					
Elderly (≥65)					
2013	5927	45	16 (4)	5–31	15 (13, 19)
2014	6633	47	18 (5)	8–43	18 (15, 21)
2015	6835	47	19 (5)	6–33	19 (16, 22)
2013–2015	19,395	46	18 (5)	5–43	17 (14, 21)
Younger (<65)					
2013	7199	55	20 (5)	9–34	20 (16, 23)
2014	7483	53	21 (5)	6–39	20 (17, 24)
2015	7738	53	21 (6)	4–38	21 (17, 24)
2013–2015	22,420	54	20 (5)	4–39	20 (17, 24)

Percent among Total Deaths (%) is percentage of the same year; Mean = daily average number of deaths; SD = standard deviation; Range = lowest daily deaths to highest daily deaths; Q1, median, Q3 are equal to the 25%, 50% and 75% number of all values in the sample arranged from small to large, respectively; CLRD = chronic lower respiratory disease; COPD = chronic obstructive pulmonary disease; Elder = age is greater than or equal to 65 years old; Younger = age is less than 65 years old.

**Table 2:** Air pollutants and meteorological factors in Shenzhen (2013–2015).

Indicator	Mean (SD)	Range	Median (Q1, Q3)	Days of Exceeding Grade 1 Criterion *	Days of Exceeding Grade 2 Criterion **
Air pollutants					
PM_2.5_ (µg/m^3^)					
2013	40 (26)	7–137	35 (20, 53)	183	39
2014	35 (20)	7–107	31 (17, 48)	159	13
2015	30 (17)	7–111	27 (16, 41)	116	6
2013–2015	35 (22)	7–137	30 (17, 47)	458	58
PM_10_ (µg/m^3^)					
2013	62 (36)	11–182	52 (33, 83)	195	8
2014	56 (28)	12–169	49 (32, 73)	178	2
2015	49 (24)	13–174	44 (30, 63)	140	1
2013–2015	56 (30)	11–182	48 (31, 72)	513	11
SO_2_ (µg/m^3^)					
2013	12 (6)	4–55	10 (8, 15)	1	0
2014	10 (4)	4–31	9 (7, 11)	0	0
2015	9 (3)	4–19	9 (8, 11)	0	0
2013–2015	10 (5)	4–55	9 (7, 12)	1	0
NO_2_ (µg/m^3^)					
2013	49 (21)	17–134	44 (34, 59)	34	34
2014	42 (16)	15–130	39 (31, 51)	9	9
2015	40 (14)	16–128	37 (30, 47)	4	4
2013–2015	44 (18)	15–134	40 (32, 52)	47	47
CO (µg/m^3^)					
2013	1163 (257)	575–1930	1134 (963, 1350)	0	0
2014	1126 (233)	619–1759	1130 (945, 1277)	0	0
2015	897 (202)	543–1671	857 (757, 1029)	0	0
2013–2015	1062 (260)	543–1930	1034 (857, 1239)	0	0
O_3_ (µg/m^3^)					
2013	53 (24)	16–140	50 (33, 70)	17	0
2014	60 (20)	26–143	55 (44, 73)	17	0
2015	52 (22)	18–131	47 (35, 68)	8	0
2013–2015	55 (22)	16–143	51 (38, 70)	42	0
Meteorological factors					
Daily average temperature (°C)					
2013	23 (5)	10–31	24 (19, 28)		
2014	23 (6)	6–31	25 (19, 29)		
2015	24 (5)	12–33	26 (19, 28)		
2013–2015	23 (5)	6–33	25 (19, 28)		
Daily average RH (%)					
2013	75 (16)	24–100	78 (67, 87)		
2014	73 (13)	19–96	76 (67, 82)		
2015	72 (11)	28–93	73 (67, 79)		
2013–2015	73 (13)	19–100	75 (67, 82)		
Daily average atmosphere pressure (Kpa)					
2013	1005 (6)	987–1019	1005 (1001, 1011)		
2014	1006 (6)	992–1021	1006 (1000, 1011)		
2015	1006 (6)	991–1019	1006 (1001, 1011)		
2013–2015	1006 (6)	987–1020	1006 (1001, 1011)		
Daily average wind speed (m/s)					
2013	2 (1)	0–6	2 (2, 3)		
2014	2 (1)	1–5	2 (2, 3)		
2015	2 (1)	1–5	2 (2, 2)		
2013–2015	2 (1)	0–6	2 (2, 3)		
Daily rainfall (0.1 mm)					
2013	6 (15)	0–101	0 (0, 3)		
2014	5 (17)	0–188	0 (0, 1)		
2015	4 (15)	0–150	0 (0, 1)		
2013–2015	4 (16)	0–188	0 (0, 1)		
Sunshine (0.1 h)					
2013	5 (4)	0–13	6 (1, 9)		
2014	6 (4)	0–12	6 (2, 9)		
2015	5 (4)	0–12	6 (2, 9)		
2013–2015	5 (4)	0–13	6 (2, 9)		

Mean = daily average number of deaths; SD = standard deviation; Range = lowest daily deaths to highest daily deaths; Q1, median, Q3 are equal to the 25%, 50% and 75% number of all values in the sample arranged from small to large, respectively. PM_2.5_ = particulate matter with a particle size below 2.5 microns. PM_10_ = particulate matter with particle size below 10 microns. RH = relative humidity. * The number of days for average daily concentration exceeded the national grade 1 criterion of China. ** The number of days for average daily concentration exceeded the national grade 2 criterion of China.

**Table 3:** The effects of PM_2.5_ concentration on residents’ mortality without controlling other pollutants and after controlling CO or O_3_ in Shenzhen (2013–2015).

Items	Single Pollutant Model				Two-Pollutant Model				Two-Pollutant Model			
	PM_2.5_				PM_2.5_ + CO				PM_2.5_ + O_3_			
	β	StdErr	*p*	ER % (95% CI)	β	StdErr	*p*	ER % (95% CI)	β	StdErr	*p*	ER % (95% CI)
All-cause mortality												
Lag0	0.0005	0.0003	0.16	0.48 (−0.19–1.17)	0.0008	0.0004	0.05 *	0.78 (0.00–1.56)	0.0004	0.0004	0.31	0.37 (−0.35–1.10)
Lag1	0.0006	0.0003	0.07 ^@^	0.61 (−0.04–1.27)	0.0009	0.0004	0.01 ^#^	0.95 (0.23–1.68)	0.0006	0.0004	0.09 ^@^	0.61 (−0.11–1.33)
Lag2	0.0007	0.0003	0.02 *	0.74 (0.11–1.38)	0.0010	0.0004	0.00 ^#^	1.00 (0.30–1.70)	0.0008	0.0004	0.04 *	0.76 (0.05–1.48)
Lag3	0.0004	0.0003	0.26	0.36 (−0.26–0.98)	0.0006	0.0003	0.10 ^@^	0.57 (−0.10–1.26)	0.0006	0.0004	0.11	0.57 (−0.14–1.27)
Lag4	0.0004	0.0003	0.16	0.44 (−0.17–1.05)	0.0008	0.0003	0.02 *	0.79 (0.12–1.47)	0.0005	0.0004	0.20	0.46 (−0.24–1.16)
Lag5	0.0007	0.0003	0.02 *	0.71 (0.10–1.32)	0.0008	0.0003	0.02 *	0.83 (0.16–1.50)	0.0011	0.0004	0.00 ^#^	1.07 (0.37–1.77)
Lag01	0.0007	0.0004	0.06 ^@^	0.70 (−0.04–1.45)	0.0010	0.0004	0.01 *	1.05 (0.23–1.88)	0.0006	0.0004	0.14	0.62 (−0.20–1.45)
Lag02	0.0009	0.0004	0.02 *	0.93 (0.14–1.73)	0.0013	0.0004	0.00 ^#^	1.26 (0.40–2.12)	0.0009	0.0005	0.05 ^@^	0.89 (−0.02–1.81)
Lag03	0.0009	0.0004	0.03 *	0.92 (0.09–1.75)	0.0012	0.0004	0.01 ^#^	1.25 (0.36–2.14)	0.0010	0.0005	0.04 *	1.04 (0.06–2.02)
Lag04	0.0010	0.0004	0.03 *	0.97 (0.11–1.83)	0.0013	0.0005	0.01 ^#^	1.30 (0.39–2.22)	0.0011	0.0005	0.03 *	1.12 (0.09–2.16)
Non-accidental mortality												
Lag0	0.0004	0.0004	0.31	0.38 (−0.36–1.11)	0.0007	0.0004	0.08 ^@^	0.75 (−0.09–1.60)	0.0003	0.0004	0.49	0.27 (−0.50–1.06)
Lag1	0.0005	0.0004	0.15	0.51 (−0.19–1.22)	0.0009	0.0004	0.03 *	0.86 (0.08–1.65)	0.0005	0.0004	0.17	0.54 (−0.24–1.32)
Lag2	0.0006	0.0003	0.09 ^@^	0.60 (−0.09–1.29)	0.0008	0.0004	0.03 *	0.83 (0.08–1.59)	0.0007	0.0004	0.07 ^@^	0.72 (−0.05–1.50)
Lag3	0.0003	0.0003	0.32	0.34 (−0.33–1.01)	0.0006	0.0004	0.10	0.61 (−0.12–1.35)	0.0006	0.0004	0.12	0.60 (−0.16–1.36)
Lag4	0.0004	0.0003	0.18	0.45 (−0.21–1.11)	0.0009	0.0004	0.02 *	0.89 (0.16–1.62)	0.0005	0.0004	0.16	0.54 (−0.21–1.30)
Lag5	0.0007	0.0003	0.05 *	0.67 (0.01–1.33)	0.0009	0.0004	0.02 *	0.88 (0.16–1.61)	0.0011	0.0004	0.00 ^#^	1.10 (0.35–1.86)
Lag01	0.0006	0.0004	0.16	0.57 (−0.23–1.38)	0.0010	0.0005	0.03 *	0.96 (0.07–1.86)	0.0005	0.0005	0.26	0.51 (−0.37–1.41)
Lag02	0.0007	0.0004	0.09 ^@^	0.74 (−0.11–1.60)	0.0011	0.0005	0.02 *	1.10 (0.18–2.04)	0.0008	0.0005	0.11	0.79 (−0.19–1.78)
Lag03	0.0008	0.0005	0.10 ^@^	0.76 (−0.14–1.66)	0.0011	0.0005	0.02 *	1.13 (0.17–2.10)	0.0010	0.0005	0.07 ^@^	0.98 (−0.07–2.04)
Lag04	0.0008	0.0005	0.08 ^@^	0.83 (−0.09–1.76)	0.0012	0.0005	0.01 *	1.24 (0.25–2.23)	0.0011	0.0006	0.05 *	1.11 (0.01–2.23)
Accidental mortality												
Lag0	0.0012	0.0009	0.17	1.19 (−0.50–2.91)	0.0010	0.0010	0.29	1.04 (−0.89–3.01)	0.0012	0.0009	0.20	1.20 (−0.64–3.07)
Lag1	0.0013	0.0008	0.10	1.36 (−0.28–3.02)	0.0016	0.0009	0.08 ^@^	1.63 (−0.18–3.47)	0.0013	0.0009	0.15	1.36 (−0.47–3.22)
Lag2	0.0018	0.0008	0.03 *	1.81 (0.22–3.42)	0.0021	0.0009	0.02 *	2.16 (0.41–3.94)	0.0013	0.0009	0.15	1.33 (−0.48–3.17)
Lag3	0.0007	0.0008	0.36	0.73 (−0.82–2.31)	0.0006	0.0009	0.49	0.61 (−1.09–2.34)	0.0008	0.0009	0.40	0.77 (−1.02–2.60)
Lag4	0.0007	0.0008	0.39	0.67 (−0.86–2.22)	0.0005	0.0009	0.56	0.50 (−1.18–2.20)	0.0004	0.0009	0.69	0.36 (−1.41–2.17)
Lag5	0.0012	0.0008	0.14	1.16 (−0.36–2.71)	0.0008	0.0009	0.38	0.76 (−0.92–2.46)	0.0012	0.0009	0.18	1.22 (−0.56–3.03)
Lag01	0.0016	0.0009	0.09 ^@^	1.61 (−0.24–3.49)	0.0017	0.0010	0.11	1.69 (−0.35–3.78)	0.0016	0.0011	0.12	1.64 (−0.44–3.77)
Lag02	0.0022	0.0010	0.02 *	2.26 (0.30–4.25)	0.0024	0.0011	0.03 *	2.40 (0.27–4.56)	0.0020	0.0011	0.08 ^@^	2.01 (−0.26–4.33)
Lag03	0.0022	0.0010	0.03 *	2.19 (0.16–4.27)	0.0022	0.0011	0.04 *	2.24 (0.05–4.47)	0.0020	0.0012	0.10	2.03 (−0.39–4.50)
Lag04	0.0021	0.0010	0.05 *	2.08 (0.02–4.17)	0.0019	0.0011	0.08 ^@^	1.95 (−0.25–4.19)	0.0018	0.0013	0.15	1.85 (−0.64–4.40)
Total respiratory mortality												
Lag0	−0.0014	0.0014	0.30	−1.43 (−4.06–1.27)	−0.0006	0.0016	0.73	−0.55 (−3.58–2.57)	−0.0019	0.0015	0.21	−1.83 (−4.64–1.06)
Lag1	0.0004	0.0013	0.74	0.44 (−2.12–3.07)	0.0009	0.0015	0.56	0.85 (−1.98–3.77)	−0.0012	0.0015	0.43	−1.15 (−3.95–1.74)
Lag2	0.0020	0.0013	0.12	2.02 (−0.49–4.58)	0.0011	0.0014	0.41	1.14 (−1.56–3.91)	0.0018	0.0014	0.20	1.85 (−0.96–4.75)
Lag3	0.0030	0.0012	0.01 *	3.04 (0.60–5.55)	0.0026	0.0013	0.05 ^@^	2.62 (−0.04–5.34)	0.0041	0.0014	0.00 ^#^	4.17 (1.40–7.02)
Lag4	0.0021	0.0012	0.08 ^@^	2.17 (−0.25–4.65)	0.0020	0.0013	0.13	2.05 (−0.59–4.77)	0.0015	0.0014	0.28	1.53 (−1.21–4.36)
Lag5	0.0023	0.0012	0.06 ^@^	2.36 (−0.05–4.83)	0.0018	0.0013	0.18	1.79 (−0.83–4.48)	0.0019	0.0014	0.18	1.88 (−0.86–4.69)
Lag01	−0.0006	0.0015	0.69	−0.59 (−3.47–2.38)	0.0001	0.0017	0.94	0.13 (−3.08–3.43)	−0.0021	0.0017	0.22	−2.07 (−5.26–1.22)
Lag02	0.0006	0.0016	0.72	0.58 (−2.50–3.76)	0.0007	0.0017	0.68	0.70 (−2.62–4.14)	−0.0009	0.0019	0.64	−0.85 (−4.39–2.81)
Lag03	0.0018	0.0016	0.27	1.82 (−1.41–5.16)	0.0017	0.0017	0.33	1.73 (−1.69–5.28)	0.0012	0.0020	0.54	1.22 (−2.59–5.17)
Lag04	0.0024	0.0017	0.16	2.42 (−0.93–5.88)	0.0021	0.0018	0.24	2.12 (−1.40–5.77)	0.0015	0.0021	0.46	1.53 (−2.48–5.71)
Chronic lower respiratory disease mortality												
Lag0	0.0015	0.0021	0.48	1.48 (−2.54–5.66)	0.0006	0.0024	0.80	0.60 (−3.93–5.35)	0.0015	0.0022	0.50	1.47 (−2.75–5.87)
Lag1	0.0012	0.0020	0.54	1.22 (−2.65–5.24)	0.0005	0.0022	0.81	0.53 (−3.68–4.92)	0.0010	0.0022	0.66	0.96 (−3.21–5.32)
Lag2	0.0043	0.0019	0.02 *	4.43 (0.71–8.29)	0.0022	0.0020	0.28	2.20 (−1.77–6.34)	0.0056	0.0020	0.01 ^#^	5.81 (1.68–10.11)
Lag3	0.0062	0.0018	0.00 ^#^	6.38 (2.78–10.11)	0.0043	0.0019	0.03 *	4.40 (0.50–8.44)	0.0093	0.0019	0.00 ^#^	9.71 (5.60–13.97)
Lag4	0.0034	0.0018	0.06 ^@^	3.41 (−0.14–7.08)	0.0021	0.0020	0.27	2.17 (−1.66–6.16)	0.0039	0.0020	0.05 ^@^	3.93 (−0.06–8.08)
Lag5	0.0028	0.0018	0.12	2.80 (−0.74–6.46)	0.0015	0.0020	0.43	1.56 (−2.26–5.53)	0.0025	0.0020	0.22	2.51 (−1.47–6.65)
Lag01	0.0016	0.0023	0.47	1.66 (−2.74–6.26)	0.0008	0.0025	0.75	0.81 (−3.98–5.83)	0.0015	0.0024	0.54	1.52 (−3.23–6.51)
Lag02	0.0035	0.0024	0.13	3.60 (−1.08–8.50)	0.0020	0.0025	0.42	2.07 (−2.89–7.29)	0.0043	0.0026	0.10	4.36 (−0.83–9.83)
Lag03	0.0057	0.0024	0.02 *	5.84 (0.94–10.98)	0.0038	0.0026	0.14	3.87 (−1.27–9.28)	0.0079	0.0027	0.00 ^#^	8.26 (2.66–14.16)
Lag04	0.0059	0.0025	0.02 *	6.05 (1.01–11.35)	0.0036	0.0026	0.18	3.64 (−1.59–9.15)	0.0083	0.0028	0.00 ^#^	8.64 (2.75–14.86)
Chronic obstructive pulmonary disease mortality												
Lag0	−0.0011	0.0027	0.68	−1.09 (−6.10–4.19)	−0.0012	0.0030	0.69	−1.18 (−6.83–4.81)	−0.0006	0.0028	0.83	−0.61 (−5.91–4.99)
Lag1	0.0000	0.0026	0.99	−0.03 (−4.92–5.12)	0.0006	0.0028	0.84	0.56 (−4.84–6.26)	−0.0001	0.0028	0.98	−0.05 (−5.37–5.56)
Lag2	0.0052	0.0024	0.03 *	5.37 (0.55–10.41)	0.0035	0.0026	0.18	3.57 (−1.57–8.98)	0.0070	0.0026	0.01 ^#^	7.29 (1.89–12.98)
Lag3	0.0079	0.0023	0.00 ^#^	8.24 (3.53–13.17)	0.0063	0.0025	0.01 *	6.52 (1.43–11.86)	0.0122	0.0025	0.00 ^#^	13.01 (7.53–18.77)
Lag4	0.0044	0.0023	0.06 ^@^	4.46 (−0.18–9.32)	0.0029	0.0025	0.26	2.89 (−2.09–8.14)	0.0053	0.0026	0.04 *	5.40 (0.12–10.95)
Lag5	0.0052	0.0023	0.03 *	5.29 (0.61–10.20)	0.0030	0.0025	0.24	3.01 (−1.98–8.25)	0.0055	0.0026	0.03 *	5.70 (0.39–11.29)
Lag01	−0.0007	0.0029	0.82	−0.67 (−6.14–5.13)	−0.0003	0.0032	0.92	−0.33 (−6.35–6.09)	−0.0003	0.0032	0.92	−0.33 (−6.31–6.02)
Lag02	0.0024	0.0030	0.43	2.40 (−3.51–8.69)	0.0019	0.0033	0.56	1.92 (−4.40–8.65)	0.0037	0.0034	0.27	3.81 (−2.85–10.94)
Lag03	0.0057	0.0031	0.07 ^@^	5.83 (−0.47–12.53)	0.0047	0.0033	0.16	4.82 (−1.81–11.90)	0.0093	0.0036	0.01 ^#^	9.72 (2.34–17.62)
Lag04	0.0062	0.0032	0.06 ^@^	6.36 (−0.18–13.32)	0.0043	0.0034	0.20	4.42 (−2.30–11.61)	0.0106	0.0038	0.01 ^#^	11.15 (3.18–19.74)
Male												
Lag0	0.0007	0.0004	0.10	0.70 (−0.14–1.56)	0.0010	0.0005	0.05 *	0.98 (0.01–1.97)	0.0005	0.0005	0.24	0.55 (−0.36–1.46)
Lag1	0.0006	0.0004	0.14	0.62 (−0.20–1.44)	0.0009	0.0005	0.06 ^@^	0.87 (−0.03–1.78)	0.0005	0.0005	0.26	0.51 (−0.39–1.42)
Lag2	0.0010	0.0004	0.01 ^#^	1.04 (0.25–1.84)	0.0011	0.0004	0.01 *	1.09 (0.23–1.97)	0.0011	0.0005	0.02 *	1.09 (0.19–1.99)
Lag3	0.0005	0.0004	0.25	0.45 (−0.32–1.23)	0.0004	0.0004	0.39	0.37 (−0.48–1.22)	0.0008	0.0004	0.07 ^@^	0.81 (−0.07–1.70)
Lag4	0.0004	0.0004	0.35	0.36 (−0.40–1.13)	0.0005	0.0004	0.28	0.46 (−0.38–1.30)	0.0004	0.0004	0.33	0.43 (−0.44–1.31)
Lag5	0.0007	0.0004	0.09 ^@^	0.65 (−0.10–1.42)	0.0004	0.0004	0.33	0.41 (−0.42–1.25)	0.0010	0.0004	0.02 *	1.03 (0.17–1.91)
Lag01	0.0009	0.0005	0.07 ^@^	0.86 (−0.07–1.79)	0.0011	0.0005	0.03 *	1.13 (0.10–2.17)	0.0007	0.0005	0.20	0.67 (−0.36–1.71)
Lag02	0.0012	0.0005	0.02 *	1.22 (0.23–2.22)	0.0014	0.0005	0.01 ^#^	1.40 (0.34–2.48)	0.0011	0.0006	0.06 ^@^	1.11 (−0.02–2.25)
Lag03	0.0012	0.0005	0.02 *	1.21 (0.18–2.25)	0.0013	0.0006	0.02 *	1.31 (0.21–2.43)	0.0013	0.0006	0.03 *	1.36 (0.14–2.58)
Lag04	0.0012	0.0005	0.02 *	1.21 (0.15–2.27)	0.0013	0.0006	0.03 *	1.26 (0.14–2.40)	0.0014	0.0006	0.02 *	1.45 (0.19– 2.72)
Female												
Lag0	0.0002	0.0006	0.77	0.16 (−0.92–1.25)	0.0005	0.0006	0.45	0.47 (−0.76–1.73)	0.0002	0.0006	0.77	0.17 (−0.98–1.34)
Lag1	0.0006	0.0005	0.25	0.61 (−0.43–1.66)	0.0011	0.0006	0.07 ^@^	1.09 (−0.07–2.26)	0.0009	0.0006	0.14	0.86 (−0.29–2.03)
Lag2	0.0003	0.0005	0.55	0.31 (−0.71–1.34)	0.0009	0.0006	0.13	0.87 (−0.25–2.00)	0.0004	0.0006	0.53	0.37 (−0.78–1.54)
Lag3	0.0003	0.0005	0.59	0.28 (−0.72–1.28)	0.0009	0.0006	0.09 ^@^	0.95 (−0.15–2.06)	0.0003	0.0006	0.57	0.33 (−0.81–1.48)
Lag4	0.0007	0.0005	0.18	0.68 (−0.31–1.67)	0.0014	0.0006	0.01 *	1.42 (0.32–2.53)	0.0007	0.0006	0.22	0.70 (−0.43–1.85)
Lag5	0.0009	0.0005	0.06 ^@^	0.94 (−0.05–1.93)	0.0016	0.0006	0.00 ^#^	1.62 (0.52–2.73)	0.0014	0.0006	0.02 *	1.39 (0.26–2.53)
Lag01	0.0005	0.0006	0.43	0.47 (−0.70–1.66)	0.0009	0.0007	0.17	0.92 (−0.39–2.24)	0.0007	0.0007	0.32	0.67 (−0.64–1.99)
Lag02	0.0005	0.0006	0.43	0.51 (−0.74–1.78)	0.0011	0.0007	0.13	1.06 (−0.31–2.44)	0.0007	0.0007	0.31	0.75 (−0.70–2.22)
Lag03	0.0005	0.0007	0.42	0.54 (−0.77–1.86)	0.0012	0.0007	0.10 ^@^	1.20 (−0.21–2.63)	0.0008	0.0008	0.30	0.82 (−0.73–2.40)
Lag04	0.0007	0.0007	0.30	0.72 (−0.63–2.09)	0.0015	0.0007	0.05 *	1.47 (0.01–2.96)	0.0010	0.0008	0.22	1.02 (−0.62–2.68)
Elder												
Lag0	0.0006	0.0005	0.22	0.61 (−0.35–1.57)	0.0009	0.0006	0.09 ^@^	0.95 (−0.15–2.06)	0.0005	0.0005	0.31	0.54 (−0.49–1.57)
Lag1	0.0009	0.0005	0.07 ^@^	0.85 (−0.07–1.78)	0.0011	0.0005	0.03 *	1.14 (0.11–2.17)	0.0009	0.0005	0.08 ^@^	0.91 (−0.11–1.93)
Lag2	0.0011	0.0005	0.02 *	1.10 (0.20–2.00)	0.0012	0.0005	0.02 *	1.22 (0.23–2.21)	0.0010	0.0005	0.06 ^@^	0.98 (−0.04–2.01)
Lag3	0.0009	0.0004	0.04 *	0.91 (0.03–1.79)	0.0011	0.0005	0.03 *	1.09 (0.12–2.06)	0.0008	0.0005	0.12	0.78 (−0.21–1.79)
Lag4	0.0010	0.0004	0.03 *	0.98 (0.12–1.86)	0.0013	0.0005	0.01 ^#^	1.30 (0.34–2.26)	0.0010	0.0005	0.06 ^@^	0.97 (−0.02–1.96)
Lag5	0.0013	0.0004	0.00 ^#^	1.32 (0.46–2.19)	0.0013	0.0005	0.01 ^#^	1.32 (0.37–2.28)	0.0017	0.0005	0.00 ^#^	1.74 (0.75–2.74)
Lag01	0.0009	0.0005	0.08 ^@^	0.92 (−0.12–1.98)	0.0013	0.0006	0.03 *	1.26 (0.10–2.43)	0.0009	0.0006	0.12	0.91 (−0.25–2.08)
Lag02	0.0013	0.0006	0.03 *	1.27 (0.15–2.40)	0.0015	0.0006	0.01 *	1.54 (0.33–2.77)	0.0012	0.0007	0.06 ^@^	1.22 (−0.06–2.52)
Lag03	0.0014	0.0006	0.02 *	1.44 (0.27–2.62)	0.0017	0.0006	0.01 ^#^	1.70 (0.45–2.96)	0.0014	0.0007	0.05 ^@^	1.37 (−0.01–2.76)
Lag04	0.0016	0.0006	0.01 ^#^	1.57 (0.38–2.78)	0.0018	0.0006	0.00 ^#^	1.82 (0.55–3.11)	0.0015	0.0007	0.04 *	1.53 (0.10–2.99)
Younger												
Lag0	0.0004	0.0005	0.39	0.41 (−0.51–1.34)	0.0007	0.0005	0.22	0.67 (−0.39–1.74)	0.0003	0.0005	0.54	0.31 (−0.68–1.31)
Lag1	0.0004	0.0005	0.40	0.39 (−0.50–1.28)	0.0008	0.0005	0.12	0.78 (−0.21–1.78)	0.0004	0.0005	0.45	0.38 (−0.60–1.38)
Lag2	0.0004	0.0004	0.35	0.42 (−0.45–1.29)	0.0008	0.0005	0.11	0.79 (−0.17–1.75)	0.0006	0.0005	0.22	0.61 (−0.37–1.60)
Lag3	−0.0001	0.0004	0.80	−0.11 (−0.96–0.74)	0.0001	0.0005	0.77	0.14 (−0.79–1.07)	0.0005	0.0005	0.36	0.45 (−0.51–1.43)
Lag4	0.0000	0.0004	0.98	−0.01 (−0.85–0.83)	0.0004	0.0005	0.45	0.35 (−0.57–1.28)	0.0001	0.0005	0.86	0.09 (−0.87–1.06)
Lag5	0.0002	0.0004	0.65	0.19 (−0.64–1.03)	0.0004	0.0005	0.36	0.43 (−0.49–1.35)	0.0005	0.0005	0.28	0.53 (−0.43–1.49)
Lag01	0.0005	0.0005	0.33	0.51 (−0.51–1.53)	0.0009	0.0006	0.13	0.86 (−0.26–2.00)	0.0004	0.0006	0.45	0.43 (−0.69–1.57)
Lag02	0.0006	0.0005	0.26	0.62 (−0.46–1.71)	0.0010	0.0006	0.10 ^@^	0.99 (−0.18–2.18)	0.0007	0.0006	0.29	0.68 (−0.57–1.94)
Lag03	0.0004	0.0006	0.44	0.45 (−0.68–1.58)	0.0008	0.0006	0.18	0.83 (−0.38–2.05)	0.0008	0.0007	0.22	0.85 (−0.49–2.20)
Lag04	0.0004	0.0006	0.53	0.38 (−0.79–1.56)	0.0008	0.0006	0.23	0.77 (−0.48–2.02)	0.0008	0.0007	0.26	0.82 (−0.59–2.25)

^#^*p* value < 0.01; * *p* value < 0.05; ^@^ *p* value < 0.10; StdErr = standard error; ER = excess risk.

## Supplementary Materials

The following are available online at http://www.mdpi.com/1660-4601/16/3/401/s1, Figure S1: RR and 95% CI of mortality per 10 µg/m^3^ increase in PM_2.5_ concentration with different lags0–5 days prior to mortality and moving averages from day 0 to day prior to mortality lag0–lag5, lag01–lag04. CLRD = chronic lower respiratory disease; COPD = chronic obstructive pulmonary disease; Elderly = greater than or equal to 65 years old; Younger = less than 65 years old.
