# Supplementary material for: Association between PM2.5 Exposure and All-Cause, Non-Accidental, Accidental, Different Respiratory Diseases, Sex and Age Mortality in Shenzhen, China
Source: Int J Environ Res Public Health. 2019 Jan 31;16(3):401. doi: 10.3390/ijerph16030401 (PMC6388241; doi:10.3390/ijerph16030401)
Supplement: Supplementary file 1 [file ijerph-16-00401-s001.pdf]

Appendix A. Supplementary material

Association between $PM_{2.5}$ exposure and All-cause, Non-accidental, Accidental, Different Respiratory Diseases, Gender and Age Mortality in Shenzhen, China

Analysis of Generalized Additive Model

Single pollutant model was used to calculate the RR value and its 95%CI lag0-lag5, lag01-lag04 of $PM_{2.5}$ 24-hour average concentration versus mortality in Shenzhen (Figure S1-1). The results showed positive correlation between $PM_{2.5}$ 24-hour average concentration with lag1, lag2, lag5, lag01-lag04 and all-cause mortality; positive correlation between $PM_{2.5}$ 24-hour average concentration with lag2, lag5, lag02-lag04 and non-accidental mortality; positive correlation between $PM_{2.5}$ 24-hour average concentration with lag2, lag01-lag04 and accidental mortality; positive correlation between $PM_{2.5}$ 24-hour average concentration with lag3-lag5 and respiratory disease mortality; positive correlation between $PM_{2.5}$ 24-hour average concentration with lag2-lag4, lag03-lag04 and CLRD mortality; positive correlation between $PM_{2.5}$ 24-hour average concentration with lag2-lag5, lag03-lag04 and COPD mortality; positive correlation between $PM_{2.5}$ 24-hour average concentration with lag2, lag5, lag01-lag04 and male mortality; positive correlation between $PM_{2.5}$ 24-hour average concentration with lag5 and female mortality; positive correlation between $PM_{2.5}$ 24-hour average concentration with lag1-lag5, lag01-lag04 and elder mortality.

After controlling CO, the results of two-pollutant models showed positive correlation between $PM_{2.5}$ 24-hour average concentration with lag0-lag5, lag01-lag04 and all-cause mortality; positive correlation between $PM_{2.5}$ 24-hour average concentration with lag0-lag2, lag4-lag5, lag01-lag04 and non-accidental mortality; positive correlation between $PM_{2.5}$ 24-hour average concentration with lag1-lag2, lag02-lag04 and accidental mortality; positive correlation between $PM_{2.5}$ 24-hour average concentration with lag3 and respiratory disease mortality, CLRD mortality, COPD mortality; positive correlation between $PM_{2.5}$ 24-hour average concentration with lag0-lag2, lag01-lag04 and male mortality; positive correlation between $PM_{2.5}$ 24-hour average concentration with lag1, lag3- lag5, lag03-lag04 and female mortality; positive correlation between $PM_{2.5}$ 24-hour average concentration with lag0-lag5, lag01-lag04 and elder mortality; with lag02 and younger mortality (Figure S1-2).

After controlling O_3, the results of two-pollutant models showed positive correlation between $PM_{2.5}$ 24-hour average concentration with lag1, lag2, lag5, lag02-lag04 and all-cause mortality; positive correlation between $PM_{2.5}$ 24-hour average concentration with lag2, lag5, lag03-lag04 and non-accidental mortality; positive correlation between $PM_{2.5}$ 24-hour average concentration with lag02 and accidental mortality; positive correlation between $PM_{2.5}$ 24-hour average concentration with lag3 and respiratory disease mortality; positive correlation between $PM_{2.5}$ 24-hour average concentration with lag2-lag4, lag03-lag04 and CLRD mortality;

positive correlation between $PM_{2.5}$ 24-hour average concentration with lag2-lag5, lag03, lag04 and COPD mortality; positive correlation between $PM_{2.5}$ 24-hour average concentration with lag2, lag3, lag5, lag02-lag04 and male mortality; positive correlation between $PM_{2.5}$ 24-hour average concentration with lag5 and female mortality; positive correlation between $PM_{2.5}$ 24-hour average concentration with lag1, lag2, lag4, lag5, lag02-lag04 and elder mortality (Figure S1-3). After controlling O_3, the positive correlation between $PM_{2.5}$ concentration and CLRD, $PM_{2.5}$ concentration and COPD mortality were all unchanged.

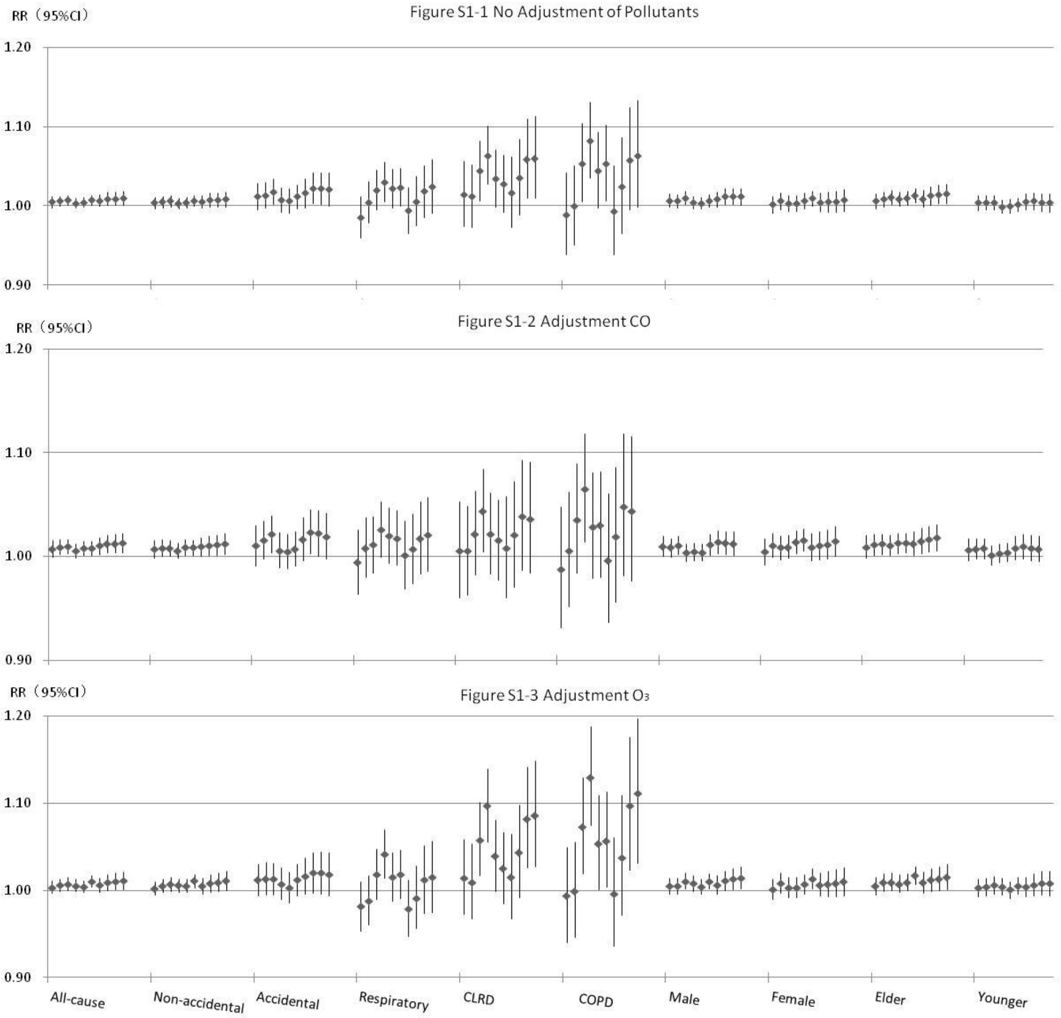

Figure S1. RR and 95%CI of mortality per 10μg/m^3 increase in $PM_{2.5}$ concentration with different lags0-5 days prior to mortality and moving averages from day 0 to day prior to mortality lag0-lag5, lag01-lag04). CLRD = chronic lower respiratory disease; COPD = chronic obstructive pulmonary disease; Elder = greater than or equal to 65 years old; Younger = less than 65 years old.
